# Corrigendum to: NGF protects neuroblastoma cells against β‐amyloid induced apoptosis via the Nrf2/HO‐1 pathway

**DOI:** 10.1002/2211-5463.12782

**Published:** 2020-01-14

**Authors:** Rui Su, Wei Su, Qian Jiao

**Affiliations:** ^1^ Department of Neurosurgery Luoyang Central Hospital Affiliated to Zhengzhou University China; ^2^ Department of Intensive Care Unit Sir Run Run Shaw Hospital Affiliated by Zhejiang University School of Medicine Hangzhou China; ^3^ Department of Anesthesia Surgery Sanmenxia Central Hospital China

The correspondence address of the paper by Su *et al.* 1 was incorrect. The correct correspondence address is provided here.

**Correspondence**

R. Su, Department of Neurosurgery, Luoyang Central Hospital Affiliated to Zhengzhou University, No. 288, Zhongzhou Middle Road, Xigong District, Luoyang, Henan 471002, China

Tel: +86 037963892086

E‐mail: ruisu5434664@163.com
